# The EPO-FGF23 Signaling Pathway in Erythroid Progenitor Cells: Opening a New Area of Research

**DOI:** 10.3389/fphys.2019.00304

**Published:** 2019-03-26

**Authors:** Annelies J. van Vuren, Carlo A. J. M. Gaillard, Michele F. Eisenga, Richard van Wijk, Eduard J. van Beers

**Affiliations:** ^1^Van Creveldkliniek, Department of Internal Medicine and Dermatology, University Medical Center Utrecht, Utrecht University, Utrecht, Netherlands; ^2^Department of Internal Medicine and Dermatology, University Medical Center Utrecht, Utrecht University, Utrecht, Netherlands; ^3^Department of Internal Medicine, Division of Nephrology, University Medical Center Groningen, University of Groningen, Groningen, Netherlands; ^4^Department of Clinical Chemistry and Haematology, University Medical Center Utrecht, Utrecht University, Utrecht, Netherlands

**Keywords:** FGF23, erythropoietin, anemia, osteoporosis, red blood cells

## Abstract

We provide an overview of the evidence for an erythropoietin-fibroblast growth factor 23 (FGF23) signaling pathway directly influencing erythroid cells in the bone marrow. We outline its importance for red blood cell production, which might add, among others, to the understanding of bone marrow responses to endogenous erythropoietin in rare hereditary anemias. FGF23 is a hormone that is mainly known as the core regulator of phosphate and vitamin D metabolism and it has been recognized as an important regulator of bone mineralization. Osseous tissue has been regarded as the major source of FGF23. Interestingly, erythroid progenitor cells highly express FGF23 protein and carry the FGF receptor. This implies that erythroid progenitor cells could be a prime target in FGF23 biology. FGF23 is formed as an intact, biologically active protein (iFGF23) and proteolytic cleavage results in the formation of the presumed inactive C-terminal tail of FGF23 (cFGF23). FGF23-knockout or injection of an iFGF23 blocking peptide in mice results in increased erythropoiesis, reduced erythroid cell apoptosis and elevated renal and bone marrow erythropoietin mRNA expression with increased levels of circulating erythropoietin. By competitive inhibition, a relative increase in cFGF23 compared to iFGF23 results in reduced FGF23 receptor signaling and mimics the positive effects of FGF23-knockout or iFGF23 blocking peptide. Injection of recombinant erythropoietin increases FGF23 mRNA expression in the bone marrow with a concomitant increase in circulating FGF23 protein. However, erythropoietin also augments iFGF23 cleavage, thereby decreasing the iFGF23 to cFGF23 ratio. Therefore, the net result of erythropoietin is a reduction of iFGF23 to cFGF23 ratio, which inhibits the effects of iFGF23 on erythropoiesis and erythropoietin production. Elucidation of the EPO-FGF23 signaling pathway and its downstream signaling in hereditary anemias with chronic hemolysis or ineffective erythropoiesis adds to the understanding of the pathophysiology of these diseases and its complications; in addition, it provides promising new targets for treatment downstream of erythropoietin in the signaling cascade.

## Introduction

At a concentration of 5 million red blood cells (RBC) per microliter blood, RBCs are the most abundant circulating cell type in humans (Eggold and Rankin, 2018). Normal erythropoiesis yields 200 billion RBCs every day, an equivalent of 40 mL of newly formed whole blood (Muckenthaler et al., 2017). Regulation of erythropoiesis in the bone marrow (BM) microenvironment depends on systemic and local factors controlling differentiation, proliferation and survival of the erythroid progenitor cells (EPC). Inherited RBC abnormalities might result in chronic hemolysis with an increased erythropoietic drive, or ineffective erythropoiesis, thereby challenging the erythropoietic system. Systemic erythropoietin (EPO) production plays a critical role in maintaining erythropoietic homeostasis under physiologic and pathologic conditions (Eggold and Rankin, 2018). Increasing evidence links EPO and erythropoiesis to skeletal homeostasis (Eggold and Rankin, 2018). First, there is a longstanding observation that patients with hemolysis have increased risk of skeletal pathology such as osteoporosis and osteonecrosis (Taher et al., 2010; Haidar et al., 2012; Eggold and Rankin, 2018; van Straaten et al., 2018). Second, removal of osteoblasts in mice resulted in increased loss of erythroid progenitors in the BM, followed by decreased amounts of hematopoietic stem cells with recovery after reappearance of osteoblasts, pointing to a critical role of osteoblasts in hemato- and erythropoiesis (Visnjic et al., 2004).

Erythropoietin, the core regulator of erythropoiesis, is an important regulator of fibroblast growth factor 23 (FGF23) production and cleavage (Clinkenbeard et al., 2017; Flamme et al., 2017; Daryadel et al., 2018; Hanudel et al., 2018; Rabadi et al., 2018; Toro et al., 2018). FGF23 is originally known as a bone-derived hormone and key player in phosphate and vitamin D metabolism. FGF23 seems to provide a link between bone mineralization and erythropoiesis (Clinkenbeard et al., 2017; Eggold and Rankin, 2018). FGF23 was first discovered as a regulator of phosphate metabolism, due to the association between hereditary phosphate wasting syndromes and *FGF23* mutations (ADHR Consortium, 2000). FGF23 induces phosphaturia, directly suppresses parathyroid hormone and the amount of 1,25(OH)_2_D_3_ (active vitamin D) (Shimada et al., 2004; Quarles, 2012). FGF23 is secreted by osteocytes in response to vitamin D, parathyroid hormone and elevated levels of serum phosphate. Due to important alterations in phosphate balance in chronic kidney disease (CKD), most research on FGF23 up until now was focused on CKD (see section “EPO, Iron, CKD, and Inflammation Are Important Regulators of iFGF23 Cleavage”) (Kanbay et al., 2017). However, a new, important role for FGF23 seems to exist as regulator of erythropoiesis.

**FIGURE 1:**
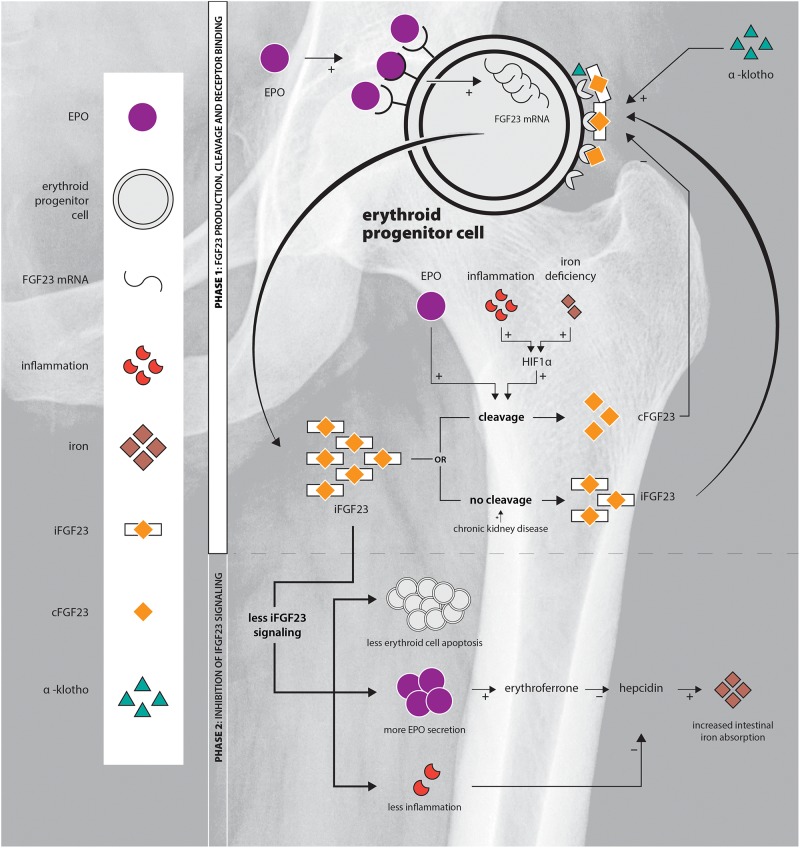
Schematic overview of the EPO-FGF23 signaling pathway in the erythroid lineage in the BM. Phase 1 displays FGF23 production, the secretory process and FGFR binding; phase 2 summarizes the effects of inhibition of iFGF23 signaling.

Here, we review the interplay of EPO and FGF23 in the erythroid cells of the BM. We discuss that the action of FGF23 not only depends on the amount of intact FGF23 available, but also on the amount of FGF23 cleavage which is an important factor determining its efficacy. Elucidation of the role of the EPO-FGF23 signaling pathway in hereditary anemia and chronic hemolytic diseases will add to the understanding of the pathophysiology of the diseases, of bone mineralization disorders complicating chronic hemolytic diseases, and might provide new targets for treatment downstream of EPO. An overview of FGF23 production, cleavage and signaling is provided in Figure 1.

## Anemia and the EPO Signaling Cascade

Erythropoietin production by renal interstitial cells, and in a smaller amount by hepatocytes, plays a critical role in maintaining erythropoietic homeostasis. The primary physiological stimulus of increased *EPO* gene transcription is tissue hypoxia, which can augment circulating EPO up to a 1000-fold in states of severe hypoxia (Jelkmann, 1992; Ebert and Bunn, 1999). Under hypoxic conditions, *EPO* transcription is augmented by binding of hypoxia inducible factor (HIF)-2 to the *EPO* gene promoter. Under normoxic conditions prolyl hydroxylases (PHD) hydroxylate HIF1α and HIF2α, which associate with the von Hippel-Lindau tumor suppressor protein, targeting this complex for proteasomal degradation. Low iron or oxygen conditions inhibit hydroxylation by PHD2 (Ebert and Bunn, 1999; Schofield and Ratcliffe, 2004). EPO exerts its effect on early erythroid progenitors via the EPO receptor (EPOR), with a peak receptor number at the CFU-E (Colony Forming Unit-Erythroid) stage and a decline until absence of the receptor in late basophilic erythroblasts. EPOR signaling results in survival, proliferation, and terminal differentiation (Krantz, 1991; Muckenthaler et al., 2017; Eggold and Rankin, 2018).

Besides kidney and liver, EPO expression has also been reported in brain, lung, heart, spleen, and reproductive organs. Besides kidney and liver, only EPO produced by the brain was capable to functionally regulate erythropoiesis (Weidemann et al., 2009; Haase, 2010). More recently, it was discovered that local production of EPO by osteoprogenitors and osteoblasts in the BM microenvironment, under conditions of constitutive HIF stabilization, results in selective expansion of the erythroid lineage (Rankin et al., 2012; Eggold and Rankin, 2018). The role of osteoblastic EPO in the BM microenvironment under physiologic conditions is still under investigation (Shiozawa et al., 2010). The amount of circulating EPO is normal or elevated in most forms of hereditary anemia, although the amount is often relatively low for the degree of anemia (Caro et al., 1979; Rocha et al., 2005; Zeidler and Welte, 2007). EPO levels were generally elevated in β-thalassemia patients with large interpatient differences partly related to age (Sukpanichnant et al., 1997; O’Donnell et al., 2007; Singer et al., 2011; Butthep et al., 2015; Schotten et al., 2017). Sickle cell disease (SCD) patients had elevated serum EPO concentrations ranging from the low end of expected for the degree of anemia to lower than expected (Pulte et al., 2014; Karafin et al., 2015). Off-label application of recombinant EPO (rhEPO) has been tried in selected patients to reduce transfusion requirements and improve quality of life. Responses varied and were unpredictable (Zachee et al., 1989; Singer et al., 2011; Fibach and Rachmilewitz, 2014; Han et al., 2017). Insight in components downstream of EPO in its signaling cascade might lead to insights in the EPO responsiveness in individual patients. FGF23 has shown to be one of those downstream components directly affecting erythropoiesis and providing feedback on EPO production, as outlined in Section “Blockade of iFGF23 Signaling Results in More Erythropoiesis.”

## Erythroid Progenitors Express FGF23 in Response to EPO

Osseous tissue has been regarded as the major source of FGF23. Selective deletion of *FGF23* in early osteoblasts or osteocytes in a murine model demonstrated that both cell types significantly contribute to circulating FGF23. However, FGF23 was still detectable in serum after deletion of the *FGF23* gene in both osteoblasts and osteocytes: other, non-osseous, tissues contribute to circulating FGF23 (Clinkenbeard et al., 2016). It was shown that BM, specifically the early erythroid lineage, does significantly contribute to total circulating FGF23. In wild-type (WT) mice treated with marrow ablative carboplatin followed by a 3-day course of rhEPO, serum FGF23 was 40% lower compared to controls (Clinkenbeard et al., 2017). In WT mice, baseline FGF23 mRNA in BM was comparable with osseous tissue, but the amount of FGF23 protein in BM tissue was significantly higher. Hematopoietic stem cells and EPCs, including BFU-E (Burst Forming Unit-Erythroid), CFU-E and proerythroblasts, showed more than fourfold higher amounts of FGF23 mRNA compared with whole BM including lineage specific cells. FGF23 mRNA was shown to be transiently expressed during early erythropoiesis (Toro et al., 2018). EPCs do express FGF23 mRNA under physiologic conditions, however significant increases are observed in response to EPO (Clinkenbeard et al., 2017; Daryadel et al., 2018; Toro et al., 2018). RhEPO induced FGF23 mRNA expression in BM cells 24 h after injection (Daryadel et al., 2018). Indirect immunofluorescence staining with anti-mouse FGF23 antibodies and lineage specific markers showed intense staining of erythroid progenitors and mature erythroblasts (CD71^+^ cells) of EPO-treated mice compared to controls (Daryadel et al., 2018).

Thus, erythroid cells of the BM significantly contribute to FGF23 production and FGF23 production is increased in response to EPO. As will be discussed in Sections “FGF23 Signaling Is Regulated by Cleavage of Intact FGF23” and “EPO, Iron, CKD, and Inflammation Are Important Regulators of iFGF23 Cleavage,” the amount of cleavage of FGF23 is equally important and EPO has a strong effect on this as well.

## FGF23 Signaling is Regulated by Cleavage of Intact FGF23

FGF23 is formed as a full-length, biologically active protein (iFGF23). Intact FGF23 is cleaved into two fragments: the inactive N-terminal fragment of FGF23 fails to co-immunoprecipitate with FGFR (FGF receptor) complexes, which suggests that the C-terminal fragment (cFGF23) mediates binding to the FGFR (Goetz et al., 2007, 2010; Courbebaisse et al., 2017). Only intact FGF23 (iFGF23) suppresses phosphate levels in mice through the FGF receptor 1 (FGFR1) (Shimada et al., 2002; Wolf and White, 2014). cFGF23 competes with iFGF23 for binding to the FGFR, and thereby antagonizes iFGF23 signaling in mice and rats (Goetz et al., 2010; Agoro et al., 2018). Treatment with cFGF23 increased the number of early and terminally differentiated BM erythroid cells and the colony forming capacity of early progenitors to the same amount as rhEPO. These data suggest that the outcome of rhEPO treatment resembles the effects of more cFGF23. Recently, it was shown that the cFGF23 fragment itself was able to induce heart hypertrophy in SCD patients (Courbebaisse et al., 2017), probably via FGFR4 and independent from a costimulatory signal (see section “Presence of α-Klotho Is Essential for Normal Erythropoiesis”) (Faul et al., 2011).

Currently, two assays are available to measure iFGF23 and cFGF23: one assay that detects the C-terminal of FGF23 which measures both cFGF23 and (full-length) iFGF23 (Immunotopics/Quidel) and one assay that only detects iFGF23 (Kainos Laboratories) (Hanudel et al., 2018). Serum half-life time is approximately identical for both iFGF23 and cFGF23 ranging from 45 to 60 min (Khosravi et al., 2007).

So, although still subject of debate, proteolytic cleavage of iFGF23 seems to abrogate its activity by two mechanisms: reduction of the amount of iFGF23 and generation of an endogenous inhibitor, cFGF23 (Goetz et al., 2010). Therefore, measurement of both iFGF23 and cFGF23 is important: alterations in the iFGF23 to cFGF23 ratio lead to alterations of iFGF23 signaling efficacy.

**FIGURE 2:**
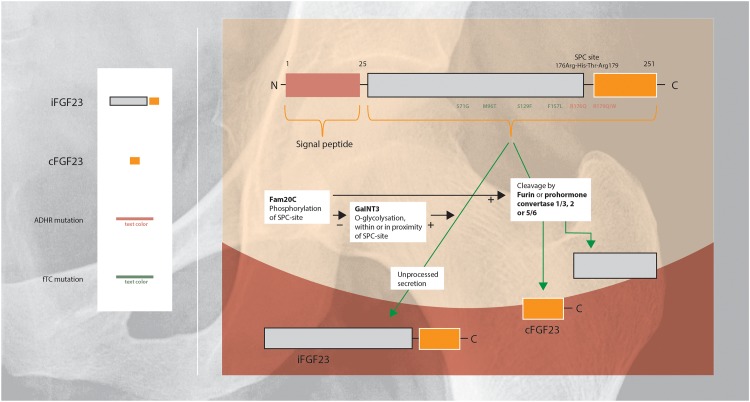
Schematic overview of the regulation of FGF23 protein cleavage and secretion (Shimada et al., 2001; Saito and Fukumoto, 2009; Huang et al., 2013; Luo et al., 2019). FGF23 harbors a naturally-occurring proteolytic site at Arg176-XX-Arg179. O-Glycosylation within or in the proximity of this SPC-site of FGF23 by GalNT3 results in increased secretion of intact FGF23. Phosphorylation of the SPC-site by Fam20C indirectly promotes FGF23 cleavage by blocking O-glycosylation. ADHR is caused by mutations near the proteolytic site, that impairs proteolytic inactivation of FGF23 resulting in high levels of iFGF23 (Arg176Gln or Arg179Gln/Trp). FTC, is an autosomal recessive disorder, resulting from mutations in the FGF23 gene which lead to destabilization of the tertiary structure of FGF23 and rendering it susceptible to degradation (Ser71Gly, Met96Thr, Ser129Phe, and Phe157Leu). FTC, familial tumoral calcinosis; ADHR, autosomal dominant hypophosphatemic rickets.

Regulation of FGF23 secretion includes intracellular processing in the Golgi apparatus in which iFGF23 is partially cleaved within a highly conserved sutilisin-like proprotein convertase (SPC)-site by furin or prohormone convertase 1/3, 2, and 5/6 (Figure 2). Cleavage of iFGF23 generates two fragments: the C- and N-terminal peptide fragments (20 and 12 kDa) (Benet-Pages et al., 2004; Tagliabracci et al., 2014; Yamamoto et al., 2016). Competition between phosphorylation and O-glycosylation of the SPC-site in the secretory pathway of FGF23 is an important regulatory mechanism of cleavage (Tagliabracci et al., 2014). Secretion of iFGF23 requires O-glycosylation: the glycosyltransferase N-acetylgalactosaminyltransferase 3 (GalNT3) selectively exerts O-glycosylation of amino acid residues within or in the proximity of the SPC-site and blocks cleavage of iFGF23 (Kato et al., 2006). In contrast, phosphorylation of the SPC-site promotes FGF23 proteolysis indirectly by blocking O-glycosylation. The kinase Fam20C phosphorylates iFGF23 within the SPC-site, consequently reduces glycosylation and subsequently facilitates iFGF23 cleavage (Yamamoto et al., 2016).

Summarizing, a proportion of synthesized iFGF23 will be cleaved intracellularly before secretion, the amount of intracellular cleavage is determined by competition between glycosylation (GalNT3) and phosphorylation (Fam20C) (Martin et al., 2012; Tagliabracci et al., 2014; Yamamoto et al., 2016). Various factors regulate post-translational modification, these are described in Section “EPO, Iron, CKD, and Inflammation Are Important Regulators of iFGF23 Cleavage.”

## EPO, Iron, CKD, and Inflammation are Important Regulators of iFGF23 Cleavage

Erythropoietin, iron, inflammation, and CKD have been identified as modifiers of iFGF23 cleavage. Notably, all these factors might co-exist in patients with hereditary anemia. The amount of cleavage is determined by alterations in GalNT3 and furin. Furin plays an important role in regulation of FGF23 cleavage in iron deficiency and inflammation (Silvestri et al., 2008; David et al., 2016), whereas under conditions of high EPO GalNT3 inhibition might augment cleavage (Hanudel et al., 2018).

### Erythropoietin

Several studies report alterations of iFGF23 and cFGF23 after administration of rhEPO or under high endogenous EPO conditions, a summary is provided in the Table 1 (Clinkenbeard et al., 2017; Flamme et al., 2017; Daryadel et al., 2018; Hanudel et al., 2018; Rabadi et al., 2018; Toro et al., 2018). Most experiments were carried out in animal models (rats and mice). Less information is available about the influence of EPO on the iFGF23/cFGF23 ratio in man.

**Table 1:** Overview studies on the effects of erythropoietin (EPO) on FGF23.

Study	Model	rhEPO	iFGF23/cFGF23
**Studies in animals**		
Clinkenbeard et al., 2017, pp e427–e430	WT C57BL/6 mice	Three-day regimen with increasing doses rhEPO	Max. ±40x increase in serum cFGF23; ±2x increase in serum iFGF23. Increases in cFGF23 in dose-dependent way.
Rabadi et al., 2018, pp F132–F139	C57BL/6 mice with and without 10% loss of total blood volume	None	6 h: ± 4x increase in plasma cFGF23; no increase in iFGF23. cFGF23 values remained increased 48 h after blood loss.
Flamme et al., 2017, p. e0186979	Male Wistar rats	Single injection rhEPO	4–6 h: >10x increase in plasma cFGF23 (extrapolated); ±2x increase in plasma iFGF23 (extrapolated).
		Single injection high dose HIF-PH inhibitor	4–6 h: comparable with rhEPO. Pretreatment anti-EPO: cFGF23 response almost absent.
Toro et al., 2018	WT C57BL/6 mice	Single injection rhEPO	4 h: ±4x increase in plasma cFGF23; ± 2.5x increase in plasma iFGF23.
	Sprague-Dawley rats, hemorrhagic shock with 50–55% loss of total blood volume	None	24 h: ±5x increase in plasma cFGF23; ± 3.5x increase in plasma iFGF23.
Daryadel et al., 2018	WT C57BL/6 mice	Single injection rhEPO	24 h: ± 2x increase in plasma cFGF23; no increase in plasma iFGF23.
		4-day regimen rhEPO	4 days: increase in cFGF23 and iFGF23.
Hanudel et al., 2018	WT C57BL/6 mice with and without 0.2% adenine diet-induced CKD	Single injection rhEPO	6 h: *non-CKD* cFGF23 207→ 3289 pg/mL; *CKD* cFGF23 2056→ 9376 pg/mL. *Non-CKD* iFGF23 187→ 385 pg/mL; C*KDI* no significant rise in iFGF23.
	Transgenic Tg6 mice overexpressing human EPO	Transgenic EPO overexpression	cFGF23 *WT* 340 pg/mL; *Tg6* 3175 pg/mL. iFGF23 *WT* 317 pg/mL, *Tg6* 589 pg/mL.
**Studies in man**		
Clinkenbeard et al., 2017, pp e427–e430	4 patients with unexplained anemia	Single injection rhEPO	6–18 h: ±2x increase in serum cFGF23; ±1.5x increase in serum cFGF23.
Rabadi et al., 2018, pp F132–F139	131 patients admitted to ICU, categorized based on number of RBC transfusions in 48 h before admission	None	Number of blood transfusions was associated with plasma cFGF23.
Daryadel et al., 2018	28 healthy volunteers	Single injection rhEPO	24 h: significant increase in plasma cFGF23; plasma iFGF23 unchanged.
Hanudel et al., 2018	680 adult kidney transplant patients	None	Higher EPO values were significantly associated with increased cFGF23 and not with iFGF23; independent of renal function.


In all animal studies one single injection or multiple-day regimen of rhEPO resulted in a significant increase in circulating cFGF23 (Clinkenbeard et al., 2017; Flamme et al., 2017; Daryadel et al., 2018; Hanudel et al., 2018; Toro et al., 2018). Increases in iFGF23 were less pronounced (Flamme et al., 2017; Hanudel et al., 2018; Toro et al., 2018), or absent (Daryadel et al., 2018), after a single injection of rhEPO. Multiple-day regimens resulted in small rises in iFGF23, less pronounced than the increase in cFGF23 (Clinkenbeard et al., 2017; Daryadel et al., 2018). EPO directly increased *FGF23* gene expression in murine hematopoietic cells (Flamme et al., 2017). Treatment of mice with an hematopoietic equipotent dose of a HIF-proline hydroxylase inhibitor (HIF-PH inhibitor) also led to a significant rise in plasma cFGF23, without an increase in circulating iFGF23. Increases in FGF23 expression after HIF-PH inhibitor treatment were mediated indirectly via EPO, as pre-administration of anti-EPO antibodies opposed upregulation of circulating FGF23 (David et al., 2016; Flamme et al., 2017).

Effects of overexpression of endogenous EPO were investigated in a transgenic human EPO-overexpressing murine model. Results were in line with responses on rhEPO in mice: circulating cFGF23 and iFGF23 were significantly higher in EPO-overexpressing mice than in WT mice (Hanudel et al., 2018). Acute blood loss in mice, as a surrogate model for high endogenous EPO, also significantly increased circulating cFGF23, but not iFGF23 (Rabadi et al., 2018).

Only four studies (Clinkenbeard et al., 2017; Daryadel et al., 2018; Hanudel et al., 2018; Rabadi et al., 2018) explored effects of EPO on FGF23 in man. In all studies, rhEPO or a condition resulting in high endogenous EPO, increased circulating cFGF23, without (Daryadel et al., 2018; Hanudel et al., 2018) or with only minimal (Clinkenbeard et al., 2017) rise in circulating iFGF23. In a large cohort of 680 kidney transplant recipients higher EPO values were associated with increased cFGF23 values and not with iFGF23 values, independent of renal function (Hanudel et al., 2018)

Together, these data show that EPO (endogenous or exogenous) increases the total amount of circulating FGF23 (iFGF23 and cFGF23) and alters the iFGF23/cFGF23 ratio in favor of cFGF23.

It is uncertain which proteins mediate increased intracellular cleavage in the secretion pathway of iFGF23 in response to EPO. In mice, experiments investigating alterations in BM mRNA expression of GalNT3 after rhEPO injection were inconclusive (Daryadel et al., 2018). Meanwhile, in EPO-overexpressing mice, compared to WT mice, GalNT3 and prohormone convertase 5/6 mRNA expression were significantly decreased in bone and BM, no differences were observed in Fam20c and furin mRNA expression (Hanudel et al., 2018). Decreases in GalNT3 mRNA and absence of changes in furin and Fam20c mRNA expression were also observed in whole BM of mice after acute blood loss. However, the amount of GalNT3 mRNA expression in isolated erythroid precursors and mature erythroblasts (Ter119^+^ cells) of these mice was unchanged (Rabadi et al., 2018). So, decreased GalNT3 expression might increase cleavage in response to high EPO, although further study is needed to elucidate the contributory of GalNT3 and other, yet unknown, mechanisms in response to EPO.

### Iron Deficiency

Iron deficiency in WT mice resulted in a significant increase of cFGF23, with a less pronounced or even absent increase in iFGF23 (Farrow et al., 2011b; Clinkenbeard et al., 2014; David et al., 2016; Hanudel et al., 2016). Treatment of iron deficiency in CKD mice resulted in a significant decrease in whole bone FGF23 (Clinkenbeard et al., 2017). Iron deficiency induced by iron chelation stabilized pre-existing HIF1α and increased FGF23 transcription (Farrow et al., 2011b; David et al., 2016). HIF1α inhibition partially blocked elevations in total FGF23, and inhibited cleavage of iFGF23 (David et al., 2016). HIF1α stabilization under conditions of iron deficiency has been associated with upregulation of furin in liver cells (Silvestri et al., 2008).

Two large cohort studies support the relevance of the observations in mice in men. In a cohort of 2.000 pre-menopausal women serum iron was inversely correlated with cFGF23, but not with iFGF23 (Imel et al., 2016). And, associations between low iron parameters and high cFGF23 and iFGF23 values were found in a cohort of 3.780 elderly, with a more pronounced increase in cFGF23 (Bozentowicz-Wikarek et al., 2015).

Multiple studies examined the effects of distinct formulations of iron, oral and intravenous, in CKD patients on circulating cFGF23 and/or iFGF23 (Okada et al., 1983; Konjiki et al., 1994; Schouten et al., 2009a,b; Hryszko et al., 2012; Prats et al., 2013; Wolf et al., 2013; Block et al., 2015; Iguchi et al., 2015; Yamashita et al., 2017; Maruyama et al., 2018). Results have been inconclusive: interacting effects of rhEPO or endogenous high EPO might have influenced results. Moreover, the carbohydrate moieties of parenteral iron formulations themselves might lead to increased amounts of iFGF23 (Blazevic et al., 2014; Zoller et al., 2017).

In summary, iron deficiency leads to increased amounts of cFGF23 fragments. HIF1α stabilization plays an important role in upregulation of intracellular iFGF23 cleavage. Due to co-existence of anemia, erythropoiesis-related factors might influence the iron deficiency-FGF23 pathway. Observed differences in expression of proteins directly involved in the secretory process of FGF23, furin and GalNT3, suggest that EPO is not simply an intermediary between iron deficiency and FGF23: furin plays an important role in the upregulation of iFGF23 cleavage in iron deficiency, whereas EPO might act via GalNT3 inhibition as discussed in Section “Erythropoietin” (Hanudel et al., 2018).

### Chronic Kidney Disease

Circulating total FGF23 rises progressively during early and intermediate stages of CKD and reaches levels of more than 1.000-times normal in advanced CKD. Elevated iFGF23 levels are considered as a compensatory mechanism for hyperphosphatemia, however regulation of FGF23 in CKD remains incompletely understood (Fliser et al., 2007; Gutierrez et al., 2009; Hanudel et al., 2018). Elevated total FGF23 is associated with progression of CKD (Fliser et al., 2007; Isakova et al., 2011; Portale et al., 2016), left ventricular hypertrophy (Faul et al., 2011), expression of IL-6 (Singh et al., 2016), impaired neutrophil recruitment (Rossaint et al., 2016), cardiovascular morbidity (Gutierrez et al., 2009; Faul et al., 2011; Mehta et al., 2016), and overall mortality (Isakova et al., 2011; Baia et al., 2013; Eisenga et al., 2017).

Besides the role of the kidney in clearance of iFGF23, CKD has also been identified as regulator of iFGF23 cleavage. Acute bilateral nephrectomy resulted in an immediate two- until threefold increase in iFGF23 levels with concomitant increase in iFGF23/cFGF23 ratio (Mace et al., 2015). In a murine CKD model, CKD was associated with less proteolytic cleavage of iFGF23 independent of iron status (Hanudel et al., 2016). Notably, iron deficiency, high endogenous EPO, or administration of rhEPO still resulted in increased total FGF23 production and cleavage in CKD (Hanudel et al., 2018).

So, CKD is associated with increased total FGF23 and alteration of the iFGF23/cFGF23 ratio in favor of iFGF23. As CKD progresses toward end-stage renal disease, the iFGF23/cFGF23 ratio will approximate 1:1 (Smith et al., 2012). Co-existence of iron deficiency or rhEPO administration still influence FGF23 secretion in CKD.

### Inflammation

The association between FGF23 and inflammation has been reported in many diseases (Munoz Mendoza et al., 2012; Hanks et al., 2015; Holecki et al., 2015; Dounousi et al., 2016; Francis and David, 2016; Okan et al., 2016; Sato et al., 2016; Resende et al., 2017; Krick et al., 2018). Multiple inflammatory signaling pathways seem to interact closely to regulate FGF23 production and cleavage during acute or chronic inflammation. Additionally, other regulators of FGF23 expression and cleavage might develop under inflammatory conditions as inflammation-induced functional iron deficiency.

Regulation of FGF23 depends on chronicity of inflammation (David et al., 2016; Francis and David, 2016). In two murine models of acute inflammation, bone FGF23 mRNA expression and serum cFGF23 concentrations increased tenfold, without changes in iFGF23 (David et al., 2016). Increases in FGF23 mRNA were absent in the presence of NFκB (nuclear factor kappa-light-chain-enhancer of activated B cells, a canonical protein complex regulating many proinflammatory genes) inhibitor, which underlines the importance of the NFκB signaling pathway in regulation of FGF23 mRNA by pro-inflammatory stimuli (Ito et al., 2015). Co-treatment of bone cells with TNF or IL-1β and furin inhibitors resulted in increased levels of iFGF23, which suggests that increased cleavage of iFGF23 during acute inflammation is mediated by furin (McMahon et al., 2005; Ito et al., 2015; David et al., 2016). HIF1α was identified as an intermediate in FGF23 mRNA upregulation: iron deficiency and hypoxia only stabilized pre-existing HIF1α, where inflammation also led to increased cellular expression of HIF1α in bone cell lines (David et al., 2016).

Chronic inflammation resulted in increased amounts of total FGF23 with increased amounts of iFGF23. Chronic inflammation seems to exhaust or downregulate the FGF23 cleavage system (Francis and David, 2016).

In the presence of inflammation, development of functional iron deficiency (Stefanova et al., 2017), discussed in Section“Iron Deficiency,” might contribute to increased cleavage of iFGF23 (David et al., 2016). The inflammatory cytokine IL-6 promotes hepcidin transcription in hepatocytes via the IL-6 receptor and subsequent activation of JAK tyrosine kinases and signal transducer and transcription activator 3 complexes that bind to the hepcidin promotor. Additionally, activin B stimulates formation of hepcidin transcriptional complexes via the BMP (bone morphogenetic protein)/SMAD signaling pathway (Verga Falzacappa et al., 2007; Besson-Fournier et al., 2012; Canali et al., 2016; Muckenthaler et al., 2017). Hepcidin controls the inflow of iron from enterocytes, the reticuloendothelial system and hepatocytes into the circulation via regulation of the expression of iron exporter ferroportin (Ganz, 2011). Upregulation of hepcidin redistributes iron to the reticuloendothelial system at the expense of FGF23 producing cells including RBC precursor cells, osteocytes, and osteoblasts. Moreover, inflammation induces proteins that scavenge and relocate iron, including lactoferrin, lipocalin 2, haptoglobin, and hemopexin. These proteins contribute to inflammation-induced functional iron deficiency (Soares and Weiss, 2015).

Summarizing, inflammation does augment both FGF23 expression and its cleavage, by increased HIF1α expression and stabilization and increased furin activity, but also via hepcidin-induced functional iron deficiency and subsequent non-hypoxic HIF1α stabilization.

## Blockade of iFGF23 Signaling Results in More Erythropoiesis

The effects of iFGF23 signaling have been studied by direct infusion of rh-iFGF23 (Daryadel et al., 2018), and by blockage of iFGF23 signaling by knockout (Coe et al., 2014), or rh-cFGF23 injection (Agoro et al., 2018). FGF23-knockout mice displayed severe bone abnormalities, reduced lymphatic organ size, including spleen and thymus and elevated erythrocyte counts with increased RBC distribution width and reduced mean cell volume, and mean corpuscular hemoglobin (Coe et al., 2014). Knockout of the *FGF23* gene in mice resulted in a relative increase in hematopoietic stem cells, with decreased apoptosis, increased proliferative capacity of hematopoietic stem cells *in vitro* to form erythroid colonies, and an increased number of immature (pro-E, Ter119^+med^, CD71^=hi^) and mature erythroid cells (Ter119^+hi^) in BM and peripheral blood. Hematopoietic changes were also observed in fetal livers, underlining the importance of FGF23 in hematopoietic stem cell generation and differentiation during embryonic development independent of the BM microenvironment. EPO, HIF1α, and HIF2α mRNA expression were significantly increased in BM, liver and kidney of FGF23-knockout mice, and the EPO receptor was upregulated on isolated BM mature erythroid cells. On the other hand, EPO, HIF1α, and HIF2α mRNA expression in osseous tissue was decreased; which might be explained by the remarkably lower osteoblast numbers in FGF23-knockout mice. Administration of rh-iFGF23 in WT mice resulted in a rapid decrease in erythropoiesis and a significant decrease in circulating EPO. *In vitro* administration of iFGF23 to FGF23-knockout BM-derived erythropoietic cells normalized erythropoiesis, normalized HIF, and EPO mRNA abundance and normalized EPOR expression (Coe et al., 2014). Alterations of EPO expression in response to iFGF23 were also observed by others: injection of rh-iFGF23 in mice reduced kidney EPO mRNA levels with 50% within 30 min, persisting over 24 h (Daryadel et al., 2018).

Inhibition of iFGF23 signaling with rh-cFGF23 in CKD mice resulted in decreased erythroid cell apoptosis, upregulation of renal and BM HIF1α and subsequent EPO mRNA expression, elevated serum EPO levels and amelioration of iron deficiency. Inflammatory markers and liver hepcidin mRNA expression declined after iFGF23 blockage (Agoro et al., 2018). Lower hepcidin expression might have followed directly from decreases in inflammation, however, might also have resulted from increased EPO expression (Wang et al., 2017).

Interestingly, the increase in erythropoiesis after iFGF23 inhibition resembles the effects of α-klotho inhibition as outlined in Section “Presence of α-Klotho Is Essential for Normal Erythropoiesis” (Xu et al., 2017). In summary, current studies underline the importance of FGFR signaling by FGF23 for early erythropoiesis.

## Presence of α-Klotho is Essential for Normal Erythropoiesis

Murine BM erythroid cells (Ter119^+^) express the FGF23 receptors FGFR1, 2, and 4, and a small amount of FGFR3 (Coe et al., 2014). The FGFR1, that among others regulates phosphaturia, needs three components to be activated: the FGFR itself, iFGF23, and α-klotho (α-KL). α-KL, first described as an aging suppressor (Kuro-o et al., 1997), forms a complex with FGFR1 subgroup c, FGFR3 subgroup c or FGFR4 thereby selectively increasing the affinity of these FGFRs to FGF23 (Kurosu et al., 2006; Urakawa et al., 2006). α-KL simultaneously tethers FGFR and FGF23 to create proximity and stability (Chen et al., 2018). Membrane-bound α-KL is predominantly expressed in kidney, parathyroid gland and brain choroid plexus, however, shed α-KL ectodomain seems to function as an on-demand cofactor (Chen et al., 2018). There is expression of α-KL mRNA in BM, including BM erythroid cells (Ter119^+^), spleen and fetal liver cells (Coe et al., 2014; Vadakke Madathil et al., 2014). The importance of α-KL for hematopoietic stem cell development and erythropoiesis was demonstrated in α-KL-knockout mice. Knockout of the α-KL gene resulted in a significant increase in erythropoiesis with significant increases in immature pro-erythroblasts and a relatively mature fraction of erythroblasts. *In vitro* α-KL-knockout BM cells generated more erythroid colonies than BM cells of WT mice. EPO mRNA expression was significantly upregulated in α-KL-knockout mice kidney, BM and liver cells, along with upregulation of HIF1α and HIF2α (Vadakke Madathil et al., 2014). Effects of α-KL-knockout are remarkably similar to effects of iFGF23 blockade or knockout. This suggests that α-KL is indeed an essential cofactor for FGF23 signaling in the regulation of erythropoiesis. However, if the link between less α-KL and more EPO involves less iFGF23 signaling remains to be proven. Besides EPO, iron load seems to influence α-KL. Iron overload decreased renal expression of α-KL at mRNA and protein level; iron chelation suppressed the downregulation of α-KL via angiotensin II (Saito et al., 2003).

Recent studies showed that FGF23 has various effects on many tissues in an α-KL-dependent way, but might also act in an α-KL-independent way especially under pathological conditions. The mechanism by which FGF23 activates the FGFR2 independent of α-KL on leukocytes and the FGFR4 independent of α-KL on cardiomyocytes is still unclear (Grabner et al., 2015; Grabner and Faul, 2016; Rossaint et al., 2016).

In conclusion, α-KL seems to be essential for FGF23 signaling in erythropoiesis, as α-KL-knockout resembles the effects of iFGF23 blockade or knockout on erythroid cell development.

## FGF23 Expression in Hereditary Anemia

Currently, information about the abundance of the EPO-FGF23 pathway in hereditary anemia is limited to two studies: one study in β-thalassemia mice and one study in SCD patients. β-thalassemia intermedia mice are characterized by anemia, iron overload and high endogenous EPO. FGF23 mRNA expression in bone and BM of thalassemia intermedia mice were elevated, reaching expression levels of endogenous EPO-overexpressing, polycythemic mice. The amount of circulating iFGF23 was significantly elevated compared to WT mice (436 versus 317 pg/mL), although the increase in iFGF23 was small compared to the increase in total circulating FGF23 (3129 versus 340 pg/mL in WT mice) (Hanudel et al., 2018). Circulating FGF23 levels were measured in 77 SCD patients, no EPO measurements were available (Courbebaisse et al., 2017). Serum ferritin concentrations and estimated glomerular filtration rate were significantly higher in SCD patients than in the control group. Mean plasma cFGF23 concentrations were significantly higher in SCD patients than in healthy controls (563 versus 55 RU/mL). The magnitude of multiplication of cFGF23 in SCD patients compared to healthy controls was comparable with the multiplication of cFGF23 observed after rhEPO (Table 1). In 75% of the SCD patients cFGF23 values were above the upper limit of normal, whereas in only 10% of the SCD patients iFGF23 values were above the upper limit of normal. Unfortunately, the association between the iFGF23/cFGF23 ratio, EPO and the extent of erythropoiesis was not evaluated.

The first study underlines that the EPO-FGF23 pathway is upregulated in β-thalassemia intermedia and can be upregulated under iron-overloaded conditions. The second study suggests that FGF23 production and cleavage are increased in SCD, if EPO or inflammation, or another factor, is the most important driving force remains to be investigated.

The activity of the EPO-FGF23 pathway in other hereditary anemias, including BM failure syndromes, with distinct amounts of hemolysis and ineffective hematopoiesis, accompanied by distinct elevations in circulating EPO, remains to be investigated. Besides activity of the pathway, the contribution of other factors influencing FGF23 signaling in hereditary anemias, including inflammation and iron load, remains to be investigated. Moreover, the role of the individual FGFRs and α-KL in FGF23 signaling in hereditary anemia is currently unknown.

## iFGF23 Directly Impairs Bone Mineralization

The mineral ultrastructure of bone is crucial for its mechanical and biological properties. Non-collagenous proteins, as osteocalcin and osteopontin, are secreted during osteoid mineralization (Gericke et al., 2005). Loss of function of either or both osteocalcin and highly phosphorylated osteopontin significantly reduces crystal thickness and results in altered crystal shape (Poundarik et al., 2018). Tissue non-specific alkaline phosphatase (TNAP) is anchored to the membranes of osteoblasts and chondrocytes and to matrix vesicles released by both cells, and degrades pyrophosphate (PPi) to Pi. Pyrophosphate is an inhibitor of bone mineralization, and the regulation of pyrophosphate by TNAP controls continuous extracellular mineralization of apatite crystals. TNAP deficiency leads to accumulation of pyrophosphate, thereby decreasing mineralization (Rader, 2017).

FGF23 and EPO, are known regulators of bone mineralization, and are discussed in Section“Fibroblast Growth Factor 23.” Finally, we discuss the contribution of these factors to defective bone mineralization in chronic diseases of erythropoiesis.

### Fibroblast Growth Factor 23

Both gain and loss of function mutations in the *FGF23* gene result in bone mineralization disorders (Table 2). Gain of function mutations in *FGF23* cause autosomal dominant hypophosphatemic rickets (AHDR), a disease marked by severe decreased bone mineral density (Benet-Pages et al., 2005; Farrow et al., 2011a; Goldsweig and Carpenter, 2015). The metabolic mirror of ADHR is familial tumoral calcinosis, which is associated with pathologic increase of bone mineral density and is caused by loss of function mutations in the *FGF23* or *GalNT3* gene (Farrow et al., 2011a; Goldsweig and Carpenter, 2015). So, disturbances in FGF23, either primary (congenital) or secondary (e.g., in response to high EPO), ultimately result in bone mineralization deficits.

**Table 2:** FGF23-related disorders.

Disease	Locus	Inheritance pattern	Genetic defect	FGF23 function	iFGF23	cFGF23	TmP/GFR	Serum calcium	Serum phosphate	Urinary phosphate	PTH	1,25(OH)_2_D	Bone features	Erythropoiesis
ADHR (OMIM 193100)	12p13.3	AD	R176Q, R179Q/W	GoF	= or ↑	↑ or =	↓	=	↓	↑	= or ↑	= or ↓	Bone deformities including varus deformity lower extremities, rachitic rosary, craniosynostosis, short stature; bone pain, bone fractures.	IDA, or low serum iron, associated with elevated FGF23 in ADHR.
fTC (OMIM 211900)	12p13.3	AR	S71G, M96T, S129F, F157L	LoF	= or ↓	↑	↑	=	↑	↓	= or ↓	= or ↑	Tumoral calcinosis, or ectopic calcifications, hyperostosis, vascular calcifications.	Not reported.


*Summary of laboratory parameters and clinical characteristics of disorders associated with gain of function (ADHR) (ADHR Consortium, 2000; Imel et al., 2007, 2011; Huang et al., 2013; Acar et al., 2017; Clinkenbeard and White, 2017; Michalus and Rusinska, 2018; Luo et al., 2019) and loss of function (fTC) mutations (Ramnitz et al., 1993; Araya et al., 2005; Larsson et al., 2005a,b; Bergwitz et al., 2009; Huang et al., 2013; Clinkenbeard and White, 2017; Luo et al., 2019) in the *FGF23* gene. AD, autosomal dominant; ADHRs, autosomal dominant hypophosphatemic rickets; AR, autosomal recessive; FGF23, fibroblast growth factor 23; fTC, familial tumoral calcinosis; GoF, gain of function; IDA, iron deficiency anemia; LoF, loss of function; PTH, parathyroid hormone; TmP/GFR, tubular maximum reabsorption rate of phosphate per glomerular filtration rate.*

FGF23 seems to act auto- and/or paracrine in the bone environment (Murali et al., 2016b). A model has been proposed for a local role of FGF23 signaling in bone mineralization, independent of α-KL, via FGFR3. Local FGF23 signaling in osteocytes results in suppression of TNAP transcription, which leads to decreased degradation, and subsequent accumulation, of pyrophosphate and suppression of inorganic phosphate production. Both directly reduce bone mineralization. Osteopontin secretion is indirectly downregulated by FGF23 signaling: lower availability of extracellular phosphate suppresses osteopontin expression (Murali et al., 2016b). Although, acting locally, also high systemically circulating FGF23 could modulate pyrophosphate metabolism (Murali et al., 2016a,b; Andrukhova et al., 2018). Moreover, alterations in vitamin D metabolism contribute to impaired bone mineralization in response to iFGF23. 1,25(OH)_2_D_3_ inhibits bone mineralization locally in osteoblasts and osteocytes via stimulation of transcription and subsequent expression of presumably inadequately phosphorylated osteopontin (Lieben et al., 2012; Murali et al., 2016b).

So, iFGF23 signaling results directly in impaired bone mineralization via TNAP suppression. Notably, current knowledge is based on FGF23-knockout models, thereby not reflecting the interplay of iFGF23 and cFGF23 (Murali et al., 2016a,b; Andrukhova et al., 2018).

### Erythropoietin

In addition to its role in erythropoiesis, EPO regulates bone homeostasis. Mice overexpressing endogenous EPO developed severe osteopenia (Hiram-Bab et al., 2015). Treatment of WT mice with rhEPO for ten days resulted in a significant reduction in trabecular bone volume and increased bone remodeling. Similar changes in bone volume were observed after increased endogenous EPO expression due to induction of acute hemolysis (Singbrant et al., 2011; Suda, 2011). Despite these observations, the action of EPO on bone homeostasis remains controversial. Effects might be dose-dependent: supraphysiologic EPO concentrations induced mineralization (Shiozawa et al., 2010; Holstein et al., 2011; Rolfing et al., 2012; Sun et al., 2012; Betsch et al., 2014; Guo et al., 2014; Wan et al., 2014; Eggold and Rankin, 2018), whereas low endogenous overexpression or moderate exogenous doses of EPO impaired bone formation via EPOR signaling (Shiozawa et al., 2010; Singbrant et al., 2011; Hiram-Bab et al., 2015; Rauner et al., 2016). Whether excess cFGF23, in response to EPO, is capable to neutralize α-KL independent osseous signaling of iFGF23, is currently unknown. We hypothesize that supraphysiologic EPO concentrations suppress the iFGF23/cFGF23 ratio to a level where the amount of cFGF23 is sufficient to fully prevent signaling of iFGF23 by competitive inhibition at the FGFR3. This resembles the hypermineralization observed in patients with elevated cFGF23 in familial tumoral calcinosis based on a *GalNT3* mutation (Ramnitz et al., 2016).

### Bone Mineralization in Disorders of Erythropoiesis

Impaired bone mineralization, osteoporosis, is an important complication of chronic disorders affecting erythropoiesis (Valderrabano and Wu, 2018). The etiology of low bone mass is multifactorial including marrow expansion, various endocrine causes, direct iron toxicity, side effects of iron chelation therapy, lack of physical activity and genetic factors (Tzoulis et al., 2014; De Sanctis et al., 2018). In SCD and thalassemia bone abnormalities have been attributed mainly to marrow expansion (Valderrabano and Wu, 2018), although a linear correlation between circulating EPO levels and degree of bone demineralization in patients with identical diseases lacked (Steer et al., 2017). Eighty percent of adult SCD patients had an abnormal low bone mineral density (Sarrai et al., 2007), and up to 90% of β-thalassemia patients had an elevated fracture risk (Christoforidis et al., 2007; Wong et al., 2016). More recently, among children and young adults receiving regular transfusions and adequate iron chelation therapy Z-scores were within the normal range (Christoforidis et al., 2007; Wong et al., 2016). The role of transfusions in correction of bone mineral density underlines the importance of EPO signaling in the etiology of bone disease.

Currently, it is unknown what the extent is of the contribution of high EPO and subsequent lowering the iFGF23/cFGF23 ratio, to impaired bone mineralization in patients with chronic disorders of erythropoiesis. We suggest that iFGF23 excreted by BM erythroid cells might act on the surrounding osteocytes and osteoblasts in an auto- and/or paracrine way which will impair bone mineralization via TNAP suppression, subsequent pyrophosphate accumulation, and indirect downregulation of ostopontin (Murali et al., 2016a,b; Andrukhova et al., 2018). Hypothetically, rhEPO therapy in selected patients might increase EPO levels toward adequately elevated EPO levels, with further decline in the iFGF23/cFGF23 ratio, ultimately turning the balance toward increased bone mineralization.

## Summary and Future Directions

We have outlined the importance of the EPO-FGF23 signaling pathways in erythroid cell development and bone mineralization. Both the amount of iFGF23 and its cleavage product cFGF23 determine signaling capacity. Insight in the activity of the EPO-FGF23 signaling pathway in rare hereditary anemias with varies degrees of hemolysis and ineffective erythropoiesis and varying circulating EPO concentrations, will add to the understanding of the pathophysiology and bone complications of these diseases.

Currently, two therapeutic agents are under development, or already registered, interfering with the EPO-FGF23 axis: FGF23 antagonists (KRN23; a therapeutic antibody against the C-terminus of FGF23) and FGFR1 inhibitor (BGJ-398; a small molecule pan-FGF kinase inhibitor) (Luo et al., 2019). Both agents have been tested for disorders characterized by high iFGF23 concentrations: tumor-induced osteomalacia (iFGF23 secreting tumors), or x-linked hyperphosphatemia (*PHEX* mutation results in high iFGF23).

Administration of rhEPO decreases the iFGF23/cFGF23 ratio, inhibiting apoptosis in erythroid cells. However, both EPO and an increase in the absolute amount of iFGF23 impair bone mineralization. Hypothetically, application of selective iFGF23 antagonists, or cFGF23 agonists, might bypass non-FGF23 related side-effects of rhEPO by regulating a more downstream component of the EPO-FGF23 pathway.

Uncertainties exist regarding (long-term) application of FGF23 antagonists or FGFR1 inhibitors in human. Thereby, the influence of FGF23, and pharmacological manipulation of FGF23, on energy metabolism is unclear. FGF23 is along with FGF21 and FGF19, both clearly associated with energy metabolism, grouped as endocrine FGFs (Luo et al., 2019).

Moreover, iFGF23 serves as a proinflammatory paracrine factor, secreted mainly by M1 proinflammatory macrophages (Hanks et al., 2015; Holecki et al., 2015; Han et al., 2016; Agoro et al., 2018; Wallquist et al., 2018). Oxygen supply in inflamed tissues is often very limited (Imtiyaz and Simon, 2010; Eltzschig and Carmeliet, 2011). This inflammation-induced hypoxia leads to increased expression of EPOR in macrophages, suppresses inflammatory macrophage signaling and promotes resolution of inflammation (Liu et al., 2015; Luo et al., 2016). In response on EPO, a substantial increase in cFGF23 compared to iFGF23 might antagonize the pro-inflammatory effects of iFGF23 or even promote development of a M2-like phenotype, characterized by immunoregulatory capacities (Rees, 2010; Liu et al., 2015; Eggold and Rankin, 2018). Several forms of hemolytic hereditary anemias present with chronic (low-grade) inflammation, which might play an important role in the vascular complications of these diseases (Frenette, 2002; Belcher et al., 2003, 2005; Aggeli et al., 2005; Rees et al., 2010; Rocha et al., 2011; Atichartakarn et al., 2014). Theoretically, cFGF23 agonists might diminish inflammation in these patients and improve clinical outcomes.

In conclusion, although first discovered as phosphate regulator, FGF23 is an important regulator of erythropoiesis being part of the EPO-FGF23 signaling pathway. A new area of research is open to extent our knowledge about FGF23 biology beyond the kidney. Experimental research is required to identify the molecular and cellular players of the EPO-FGF23 signaling pathway and the role of the various FGFRs in erythropoiesis. Thereby, to determine the clinical relevance of the pathway in patients with alterations in erythropoiesis, we propose measuring iFGF23, cFGF23, and EPO levels in patients with various forms of dyserythropoietic or hemolytic anemia, and relating these values to inflammation, bone health and vasculopathic complications.

## Author Contributions

All authors listed have made a substantial, direct and intellectual contribution to the work, and approved it for publication.

## Conflict of Interest Statement

The authors declare that the research was conducted in the absence of any commercial or financial relationships that could be construed as a potential conflict of interest.

## Abbreviations

ADHR: autosomal dominant hypophosphatemic rickets
α-KL: α-klotho
BM: bone marrow
CKD: chronic kidney disease
EPC: erythroid progenitor cell
EPO: erythropoietin
EPOR: erythropoietin receptor
FGF: fibroblast growth factor
FGFR: fibroblast growth factor receptor
GalNT3: *N*-acetylgalactosaminyltransferase 3
HIF: hypoxia inducible factor
HIF-PHI: hypoxia inducible factor proline hydroxylase inhibitor
PHD: prolyl hydroxylase
RBC: red blood cell
rhEPO: recombinant erythropoietin
SCD: sickle cell disease
SPC: sutilisin-like proprotein convertase
TNAP: tissue non-specific alkaline phosphatase
WT: wild-type
